# In utero nanoparticle delivery for site-specific genome editing

**DOI:** 10.1038/s41467-018-04894-2

**Published:** 2018-06-26

**Authors:** Adele S. Ricciardi, Raman Bahal, James S. Farrelly, Elias Quijano, Anthony H. Bianchi, Valerie L. Luks, Rachael Putman, Francesc López-Giráldez, Süleyman Coşkun, Eric Song, Yanfeng Liu, Wei-Che Hsieh, Danith H. Ly, David H. Stitelman, Peter M. Glazer, W. Mark Saltzman

**Affiliations:** 1 Department of Biomedical Engineering, Yale University, New Haven, CT 06511 USA; 2 Department of Therapeutic Radiology, Yale University, New Haven, CT 06520 USA; 3 Department of Surgery, Yale University, New Haven, CT 06520 USA; 4 Department of Genetics, Yale University, New Haven, CT 06520 USA; 5 Yale Center for Genome Analysis (YCGA), Yale University, New Haven, CT 06477 USA; 6 Department of Neurosurgery, Yale University, New Haven, CT 06520 USA; 7 Department of Chemistry and Center for Nucleic Acids Science and Technology (CNAST), Carnegie Mellon University, Pittsburgh, Pennsylvania 15213 USA; 8 Present Address: Department of Pharmaceutical Sciences, University of Connecticut, Storrs, CT 06269 USA

## Abstract

Genetic diseases can be diagnosed early during pregnancy, but many monogenic disorders continue to cause considerable neonatal and pediatric morbidity and mortality. Early intervention through intrauterine gene editing, however, could correct the genetic defect, potentially allowing for normal organ development, functional disease improvement, or cure. Here we demonstrate safe intravenous and intra-amniotic administration of polymeric nanoparticles to fetal mouse tissues at selected gestational ages with no effect on survival or postnatal growth. In utero introduction of nanoparticles containing peptide nucleic acids (PNAs) and donor DNAs corrects a disease-causing mutation in the β-globin gene in a mouse model of human β-thalassemia, yielding sustained postnatal elevation of blood hemoglobin levels into the normal range, reduced reticulocyte counts, reversal of splenomegaly, and improved survival, with no detected off-target mutations in partially homologous loci. This work may provide the basis for a safe and versatile method of fetal gene editing for human monogenic disorders.

## Abstract (web summary)

The correction of genetic defects in utero could allow for improved outcomes of gene therapy. Here, the authors demonstrate safe delivery of nanoparticles to fetal mouse tissues, and show that nanoparticles containing peptide nucleic acids to edit the beta-globin gene are effective in a mouse model of beta-thalassemia.

## Introduction

Every year, an estimated 8 million children are born worldwide with severe genetic disorders or birth defects. Of these diseases, hemoglobinopathies are the most commonly inherited single-gene disorders, with a global carrier frequency of over 5%^1^. Depending on the severity of the disease, children affected by β-thalassemia may require lifelong transfusions or bone marrow transplantation, which can lead to serious complications such as iron overload, sepsis, or graft-versus-host disease. Recent advances in non–invasive genetic testing allow for diagnosis of genetic disorders such as thalassemia early in gestation^2^, providing a window during which genetic correction could be pursued prior to birth. In utero gene therapy thus far has focused on stem-cell transplantation and viral-mediated gene delivery [reviewed in ref. ^3,4^], methodologies that do not allow for correction of a gene in its endogenous environment. Considerable advances in gene therapy approaches have occurred, but they still face challenges associated with the use of viruses and with the risk of ectopic integration into deleterious sites in the genome, issues of particular concern for a developing fetus.

In the past decade, site-specific gene editing to correct disease-causing mutations has emerged as an attractive approach to ameliorate genetic diseases, with substantial effort directed at development of nuclease-based editing tools such as CRISPR/Cas9. As an alternative, our group has recently shown that gene correction can be coordinated efficiently and safely in postnatal animals via the intravenous or inhalational administration of polymeric, biodegradable nanoparticles (NPs) loaded with triplex-forming peptide nucleic acids (PNAs) and single-stranded donor DNAs^5–7^. The PNAs contain nucleobases supported by a modified polyamide backbone^8^ and bind to their specific genomic target site via both Watson−Crick and Hoogsteen base-pairing^9^, yielding PNA/DNA/PNA triplex structures that induce endogenous DNA repair to mediate the recombination of the donor DNA molecule containing the correct sequence and produce specific, in situ gene correction^10–12^. This process is, in part, dependent on the nucleotide excision repair and homology dependent repair pathways^10,13^ [reviewed in ref. ^12,14^].

PNAs do not readily cross the cellular membrane^15^ and are rapidly cleared within 10–30 min after intravenous or intraperitoneal administration^16^, thus, a delivery vehicle is needed to achieve in vivo gene editing. We previously demonstrated that PNA and donor DNA could be efficiently encapsulated in NPs fabricated from poly(lactic-co-glycolic acid) (PLGA), a polymer that has been approved by the FDA for numerous drug delivery applications^17^. When compared to treatment with naked oligos, PNA/DNA NP formulations led to thousands-fold higher gene editing both in vitro and in vivo^5,17^.

In earlier work, we showed that genomic correction achieved by PNA/DNA NPs leads to significant gene editing and phenotypic disease improvement in mouse models of β-thalassemia and cystic fibrosis^5–7^. Unlike gene editing technologies that rely on the activity of exogenously delivered nucleases^18,19^—such as zinc finger nucleases, TAL effector nucleases, and CRISPR/Cas9—PNA/DNA NPs can be readily administered in vivo and have been shown to have extremely low to undetectable off-target effects in the genome because the PNA editing molecules lack inherent nuclease activity^5–7^.

Here, we sought to determine the feasibility, safety, and efficacy of in utero gene editing mediated by PNA/DNA-containing NPs. We find that NPs can be delivered to multiple fetal mouse tissues intravenously, with the most pronounced accumulation in the fetal liver, the site of fetal hematopoiesis. In contrast, intra-amniotic NP delivery results in preferential NP accumulation in the fetal lung and gut at gestational ages later than 15 days. We find that both delivery approaches are minimally invasive and do not hinder fetal development, long-term survival, or reproductive potential. Using NPs loaded with next-generation, chemically modified γPNAs and DNAs for in utero treatment, we corrected a disease-causing β-thalassemia mutation in fetal mice to yield persistent postnatal amelioration of disease as measured by elevation of hemoglobin concentration, improved red blood cell morphology, decreased reticulocyte counts, and reduction of extramedullary hematopoiesis, accompanied by a clinically relevant level of editing in both fetal and adult bone marrow. Importantly, we observed a substantial long-term postnatal survival advantage for the in utero treated animals versus untreated controls, highlighting the potential for clinical translation of our work.

## Results

### Biodistribution of nanoparticles in utero

**Fig. 1:**
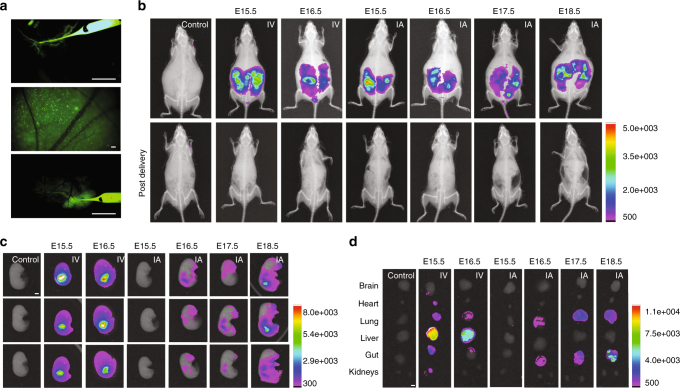
Biodistribution of poly(lactic-co-glycolic acid) (PLGA) nanoparticles (NPs) after vitelline vein (IV) or intra-amniotic (IA) delivery. **a** Stereomicroscope image of a glass micropipette injecting NPs loaded with coumarin 6 (C6, green) into the vitelline vein at E15.5 (top), NPs in E15.5 fetal circulation 3 h after vitelline vein delivery (middle), and intra-amniotic injection of C6 NPs at E16.5 (bottom) (*n* = 69 fetuses IV; *n* = 140 fetuses IA). Images are representative screen captures from full movies that are available in the supplementary materials (Supplementary Movies 1–3). Scale bars = 5 mm. **b–d** Distribution of DiD-loaded PLGA NPs 3 h after either IV or IA fetal injection in (**b**) time-dated pregnant B6 mice before delivery (top) and post surgical delivery of injected fetuses (bottom), (**c**) individual fetuses, and (**d**) fetal organs (control *n* = 9 fetuses, E15.5 IV *n* = 10 fetuses, E16.5 IV *n* = 7 fetuses, E15.5 IA *n* = 7 fetuses, E16.5 IA *n* = 8 fetuses, E17.5 IA *n* = 9 fetuses, E18.5 IA *n* = 10 fetuses). Scale bars = 2 mm

Fetal surgeons and maternal fetal medicine physicians can safely access the amniotic cavity for amniocentesis and cannulate umbilical vessels for fetal blood transfusions under ultrasound guidance as early as 18 weeks of gestation in humans^20,21^. These procedures have been used in clinical practice since the 1980’s and carry a low risk of fetal loss (~ 1%)^20,22,23^. We hypothesized that similar techniques could be used to introduce NPs safely in utero. We tested this hypothesis using PLGA NPs encapsulating fluorescent dyes. All NPs used were spherical and similar in size (~200 nm) and zeta potential (~ −25 mV) (Supplementary Fig. 1). Fluorescent NPs were administered to fetal B6 mice either intravenously via the vitelline vein, as a proxy for human umbilical vein transfusion, or directly into the amniotic cavity at gestational ages later than E15 (Fig. 1a and Supplementary Movies 1, 2). Administration of NPs to fetal mice results in particle retention within the fetuses with no detectable particle accumulation in the maternal mouse (Fig. 1b and Supplementary Figs. 2–4). As a positive control, fluorescent NPs were directly administered to the maternal circulation of a mouse pregnant with fetuses at E15.5, which results in particle accumulation within the maternal liver (Supplementary Fig. 3). Intra-vitelline vein delivery of fluorescent PLGA NPs results in widespread particle distribution throughout the fetus at both E15.5 and E16.5 with the most abundant NP accumulation in the fetal liver (Fig. 1c, d and Supplementary Fig. 2), but no accumulation in the liver of the mother. Substantial accumulation of NPs in the fetal liver is expected during development because the extraembryonic vitelline veins anastomose to form the portal circulation.

During physiologic mammalian fetal development, the fetus breaths amniotic fluid into and out of the developing lungs, providing the necessary forces to direct lung development and growth^24^. Developing fetuses also swallow amniotic fluid, which aids the formation of the gastrointestinal tract^25^. Thus, introduction of NPs into the amniotic fluid at gestational ages after the onset of fetal breathing and swallowing could result in their direct delivery to the respiratory and gastrointestinal tracts, respectively. Intra-amniotic (IA) injection of fluorescent NPs (Fig. 1a and Supplementary Movie 3) at E15.5 did not lead to any detectable particle accumulation within the fetus (Fig. 1c, d, and Supplementary Fig. 4). However, IA injection at E16.5—the expected time of onset of pronounced fetal breathing and swallowing^26^—resulted in particle accumulation in the fetal lung and gut. NP accumulation in the lung and gut was also observed after IA injections at E17.5 and E18.5, with increased intensity of NP accumulation at the later gestational ages (Fig. 1c, d and Supplementary Fig. 4).

### Safety of in utero delivery of NPs containing nucleic acids

Our gene editing approach is mediated by PLGA NPs-encapsulating PNAs and single-stranded donor DNAs^5–7,17,27^. The PNAs incorporate a tail-clamp PNA (tcPNA) reagent design, in which the Watson-Crick binding domain is extended longer than the Hoogsteen domain for increased binding affinity and specificity^28,29^. The PNAs are also substituted at the γ-backbone position with a mini-polyethylene glycol (mini-PEG) side chain to provide increased solubility and to enforce a helical pre-organization that further enhances binding affinity for the DNA target^30^. A γtcPNA/DNA pair developed to correct the β-thalassemia-causing IVS2-654 mutation in the *β-globin* gene^6^ was loaded into PLGA NPs. These NPs showed a spherical shape with sizes and zeta potentials (Supplementary Fig. 5a) and nucleic acid release profiles (Supplementary Fig. 5b) consistent with previous formulations^5–7^. The tcPNAs used for this study were synthesized with γ mini-PEG substitutions at alternating residues in the Watson–Crick-binding domain^6^. We had previously shown that PNAs containing alternating γPNAs induce gene editing at higher frequencies than unmodified PNAs both ex vivo and in vivo^6^. Partial modification with γ side chains is sufficient to pre-organize the molecules into a helical conformation that markedly increases DNA binding^31^.

**Fig. 2:**
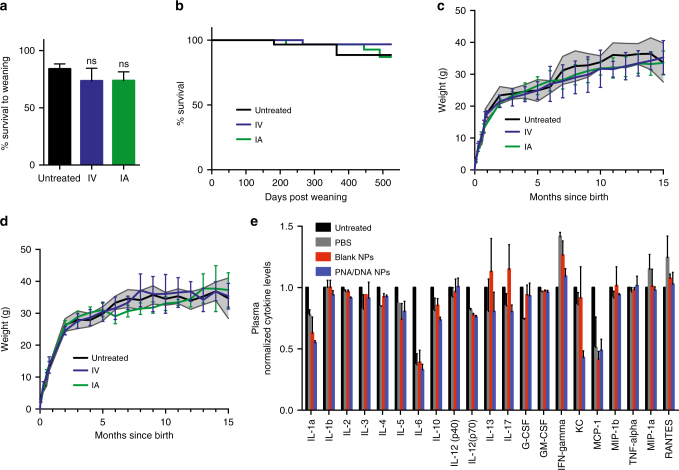
Safety of in utero nanoparticle (NP) delivery. **a** Survival to weaning (21 days) of mice injected intravenously (IV) or intra-amniotically (IA) with γtcPNA/DNA NP compared to mice subjected to sham surgery (untreated controls) (untreated *n* = 7 litters, IV *n* = 4 litters, IA *n* = 7 litters), the data are mean ± s.e.m., statistical analysis by one-way ANOVA, *p* = 0.5053. **b** Long-term survival of IV and IA NP injected mice compared to untreated controls (*n* = 15 females and *n* = 15 males for each group), statistical analysis by Log-rank Mantel-Cox test, *p* = 0.8490. **c**, **d** Weight of IV and IA NP injected (**c**) female and (**d**) male mice compared to untreated controls, (*n* = 10 for each group), gray-shaded region indicates the standard deviation of the control group. The data are shown as mean ± s.d., statistical analysis by two-way ANOVA. **e** Analysis of cytokine levels in plasma of E15.5 fetuses treated IV with PBS, blank NPs, or γtcPNA/DNA NP compared to untreated fetuses 48 h post treatment (*n* = 3 for each group), the data are mean ± s.e.m., statistical analysis by two-way ANOVA

Because this work represents the first attempt to carry out NP-mediated gene editing in utero, we initially tested the safety of injecting these PNA and DNA-containing NPs. We chose to focus first on wild-type mice to assess the impact of the NPs on growth and survival in the absence of disease. We found no significant differences in the survival of pups to weaning between those treated in utero with NPs, either intravenously or intra-amniotically, compared to pups that received sham surgery, but no in utero NP treatment, which we refer to as untreated mice (Fig. 2a). For the mice that lived to weaning, we observed no significant differences in the long-term survival between untreated mice and those that received NP treatment in utero (Fig. 2b). After pups were born and matured, we also observed no significant differences between untreated mice and those that received in utero NP treatment with respect to growth patterns or body weights (Fig. 2c, d). No gross anatomical deformities, developmental abnormalities, or tumors were observed in the mice that received in utero NP treatments (*n* = 72). Mice that had been treated with NPs in utero were able to have successful pregnancies and litters that were also free of gross abnormalities and tumors (*n* = 14 litters, *n* = 99 pups). We also measured fetal plasma cytokine levels 48 h after the IV delivery of PBS, blank NPs, and PNA/DNA NPs. There were no significant increases in levels of any of the proinflammatory cytokines measured in the NP treated groups compared to untreated fetuses (Fig. 2e), which is consistent with our previous work in adult animals^6^. For reference, administration of a small dose of lipopolysaccharide (LPS) as a positive control elicits a response in which inflammatory cytokines are elevated over 200-fold^6^.

### In utero gene correction leads to phenotypic improvement

As in the human hematopoietic system^32^, murine hematopoietic stem cells (HSCs), the target cells for gene correction in thalassemia, first emerge in the para-aortic splanchnopleure after the 7^th^ day of gestation, E7.5, and later in the aorto-gonad-mesonephros, umbilical and vitelline vessels on E10.5^33-35^. HSCs that develop in these tissues do not differentiate, but go on to seed the fetal liver on E14.5, creating a niche that is capable of supporting HSC self-renewal. Within the fetal liver, HSCs undergo a massive expansion before further seeding the spleen, thymus, and finally the bone marrow at the beginning of E16.5^34,36^. The rapid cycling and expansion of HSCs in the fetal liver contrasts with adult bone marrow HSCs, which are largely quiescent^37^. Because our prior work identified increased DNA repair and gene editing in activated stem cells^5,6^, we hypothesized that the ability to target this rapidly dividing and expanding stem cell population in the fetal liver might represent an important therapeutic opportunity for gene editing.

Given our observation that NPs injected in the vitelline vein on day E15.5 accumulate in the fetal liver during this time of rapid HSC expansion, we chose that time and administration route to test the in utero gene editing potential of PNA/DNA NPs in a transgenic mouse model of β-thalassemia. In this model, the two (*cis*) murine β-globin genes are replaced with a single copy of the human *β-globin* gene containing a β-thalassemia-associated splice site mutation in intron 2 at position 654. No homozygous *Hbb*^*th-4*^*/Hbb*^*th-4*^ mice survive postnatally. Heterozygous *Hbb*^*th-4*^*/Hbb*^*+*^ mice produce reduced amounts of mouse β-globin chains and no human β-globin, resulting in β-thalassemia marked by microcytic anemia and splenomegaly^38^.

**Fig. 3:**
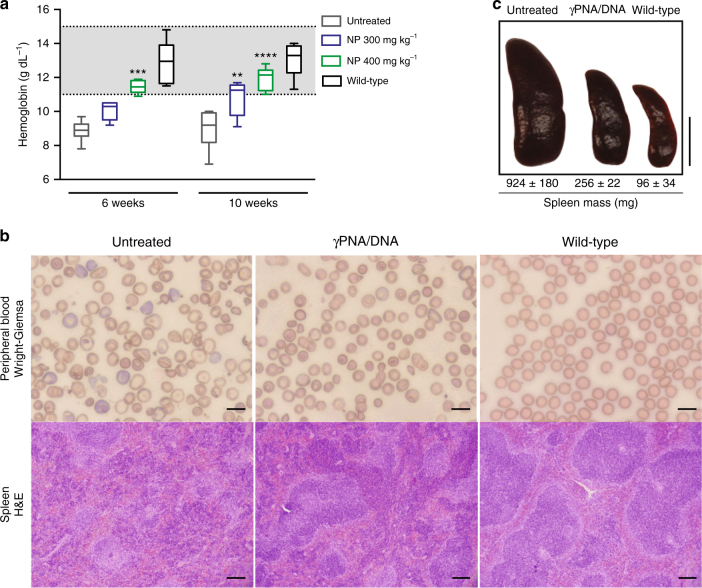
E15.5 intravenous (IV) delivery of γtcPNA/DNA nanoparticles (NPs) corrects anemia in thalassemic mice. **a** Blood hemoglobin levels of untreated *Hbb*^*th-4*^*/Hbb*^*+*^ mice, *Hbb*^*th-4*^*/Hbb*^*+*^ mice treated with γtcPNA/DNA NPs at E15.5, and wild-type B6 mice, wild-type hemoglobin range is denoted by the gray-shaded region between 11.0–15.1 g dl^−1^, (*n* = 6 for all groups), horizontal lines within the boxes indicate the median, the box indicates the first and third quartile, and the whiskers represent the range, statistical analysis by two-way ANOVA, **P < 0.01, ***P < 0.001, ****P < 0.0001. **b** Wright–Giemsa stained blood smears of γtcPNA/DNA NP treated mice 15 weeks post treatment compared to untreated *Hbb*^*th-4*^*/Hbb*^*+*^ and wild-type B6 mice (top) and H&E stained spleen sections from untreated *Hbb*^*th-4*^*/Hbb*^*+*^ mice, *Hbb*^*th-4*^*/Hbb*^*+*^ mice 15 weeks after E15.5 400 mg kg^−1^ NP treatment and wild-type mice, ×4.2 magnification (bottom). Peripheral blood scale bars = 10 μm, spleen scale bars = 150 μm. **c** Gross images of spleens from untreated *Hbb*^*th-4*^*/Hbb*^*+*^ mice, *Hbb*^*th-4*^*/Hbb*^*+*^ mice 15–30 weeks after E15.5 400 mg kg^-1^ NP treatment and wild-type mice. The spleen mass ± s.e.m. is reported for each treatment group, (untreated *n* = 7, γPNA/DNA *n* = 3, wild-type *n* = 3). Scale bar (right) = 1 cm

To test if in utero γPNA/DNA delivery could lead to gene editing and postnatal disease improvement, NPs (300 or 400 mg kg^−1^ per fetus based on an average E15.5 fetal weight of 0.45 g) were administered intravenously to each fetus via the vitelline vein at E15.5. The resulting pups were genotyped prior to weaning. The blood hemoglobin concentrations of treated heterozygous mice were measured at six and ten weeks of age. At both doses, fetuses that were treated with NPs developed into adult mice with significantly higher levels of hemoglobin than untreated β-thalassemic mice. Notably, the higher dose of γPNA/DNA NPs resulted in a greater elevation of hemoglobin concentrations, yielding values in the wild-type range at both six and ten weeks of age (Fig. 3a). The sustained elevation in postnatal hemoglobin was accompanied by a clear improvement in red blood cell (RBC) morphology on peripheral blood smear (Fig. 3b). In contrast, the peripheral smears of untreated mice continued to display anisocytosis, poiklocytosis, and an abundance of target cells, all of which are hallmarks of β-thalassemia (Fig. 3b).

**Fig. 4:**
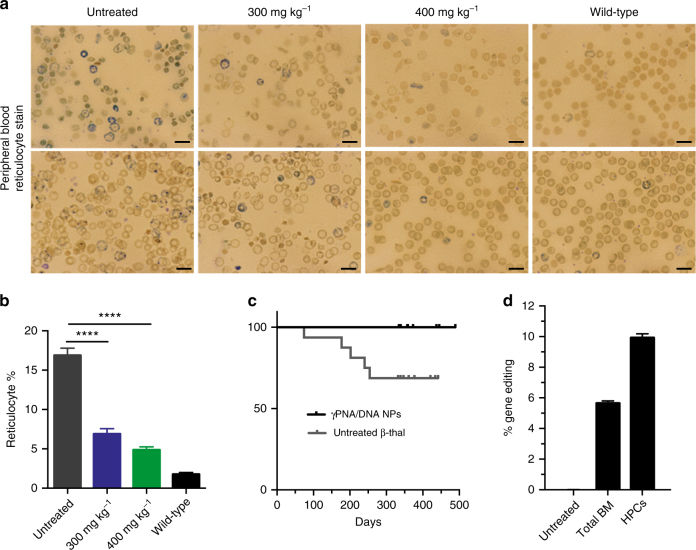
Additional evidence for correction of anemia and disease phenotype in thalassemic mice after in utero γtcPNA/DNA nanoparticle (NP) delivery. **a**, **b** Reticulotye (**a**) stains (blue) and (**b**) percentage of total red blood cells (RBCs) of *Hbb*^*th-4*^*/Hbb*^*+*^ mice 10 weeks after E15.5 intravenous (IV) γtcPNA/DNA NP treatment compared to untreated *Hbb*^*th-4*^*/Hbb*^*+*^ and wild-type B6 mice, (*n* = 6 for all groups), the data are mean ± s.e.m., statistical analysis by two-way ANOVA, ****P < 0.0001. Scale bars = 10 μm. **c** Survival of *Hbb*^*th-4*^*/Hbb*^*+*^ thalassemic mice treated in utero on day E15.5 by IV injection of γtcPNA/DNA NPs versus untreated age-matched controls (*n* = 16 for both groups), with statistical analysis by Log-rank Mantel–Cox test, *p* = 0.02. **d** Droplet digital PCR (ddPCR) quantification of gene editing in genomic DNA from total bone marrow and isolated hematopoietic progenitor cells (HPCs) collected from mice 15 weeks post-treatment (E15.5 by IV injection of γtcPNA/DNA NPs), compared to untreated *Hbb*^*th-4*^*/Hbb*^*+*^ controls, (*n* = 3), data are mean ± s.e.m

*Hbb*^*th-4*^*/Hbb*^*+*^ mice have dramatically enlarged spleens, which is consistent with the splenomegaly due to extramedullary hematopoiesis seen in patients with thalassemia^38^. In utero NP administration produced a 73% reduction in splenic weight in treated mice, compared to controls, measured in adult animals over 15 weeks after the in utero NP treatment (Fig. 3c). The observed reduction in splenomegaly correlated with improved splenic architecture in treated mice, with prominently defined red and white pulp, similar to that normally seen in wild-type (Fig. 3b). The normal demarcations between the white pulp and surrounding red pulp are blurred in the untreated *Hbb*^*th-4*^*/Hbb*^*+*^ mice (since extramedullary hematopoiesis leads to expansion of the red pulp and disruption of the white pulp). Decreased immunostaining for CD44 (stains erythroid precursors and staining decreases during terminal erythroid differentiation, but is not specific^39,40^), CD71 (specifically stains early erythroblasts^39,41^), E-cadherin (immature erythroid precursors^42^) and CD61 (megakaryocytes) in treated compared to untreated mice additionally suggests a reduction in extramedullary hematopoiesis (Supplementary Figs. 6, 7). Taken together, the reduction of splenomegaly and improvement in splenic histology pattern in treated mice further indicates alleviation of anemia. In addition to elevated hemoglobin levels, improved RBC morphology, and reduction of splenomegaly, we also found significantly reduced reticulocyte counts in the peripheral blood of treated mice (Fig. 4a, b), again indicating substantial correction of anemia.

Importantly, the mice treated with NPs in utero also showed a significant postnatal survival advantage compared to untreated controls. At 500 days after birth, in utero treated mice had 100% survival, in contrast to just 69% survival in the untreated group (Fig. 4c).

### Confirmation of in utero gene correction at the DNA level

To quantify the extent of gene editing achieved in the mice that leads to these observed postnatal improvements in anemia, we performed deep sequencing analysis on genomic DNA extracted from total bone marrow of postnatal mice at a 15 week time-point counting from the day of the in utero NP treatment. Correction of the targeted mutation was detected at a frequency of ~6% in the total bone marrow (Table 1). Deep sequencing was also used to assess off-target effects in the bone marrow by evaluating seven other genomic sites with partial homology to the binding site of the γPNA^6^. The results revealed an undetectable mutation frequency at these off-target sites in the bone marrow of treated mice (Table 1). The total measured off-target frequency combining all sites is <0.000002%.

**Table 1:** Deep sequencing analysis of targeted gene editing versus off-target effects in bone marrow cells 15 weeks after a single in utero γtcPNA/DNA nanoparticle (NP) treatment

Gene locus	Sequences of partial homology (5′ to 3′)	Size of region sequenced	Amplicons sequenced	Number modified	Frequency (%)
β-globin	TGCCCTGAAAGAAAGAGA	128	7230901	448040	6.2
Vascular cell adhesion protein precursor 1	AGCCCTGAAAGAAAGAGA	111	6280254	0	0
Polypyrimidine tract binding protein	GAACCTGAAAGAAAGAGA	101	4452674	0	0
Protocadherin fat 4 precursor	CACCCTGAAAGAAAGAAG	115	5742183	0	0
Olfactory receptor 266	AAGCCTGAAAGAAAGATT	172	6148312	0	0
Syntaxin-binding protein	AGAAATGAAAGAAAGAGA	150	6660839	0	0
Muscleblind-like protein	GGTGGTGAAAGAAAGAGA	165	5553180	0	0
Ceruloplasmin isoform	AGGACTGAAAGAAAGAGT	154	6197021	0	0
Total off-target			41034463	0	<0.000002

The top seven gene loci in the mouse genome with partial homology to the 18 bp γPNA target site in β-globin intron 2 were previously identified^6^, with the sequences as indicated. Genomic DNA from total bone marrow cells 15 weeks post NP treatment was subject to deep sequencing analysis at these loci of partial homology as well as the target locus. The size of the region sequenced around each site, the total number of amplicons sequenced and the number of amplicons with modified sequences are listed

In addition, to evaluate editing specifically in putative hematopoietic stem/progenitor cells (HSCs), we collected fetal bone marrow on day E18.5 (three days after injection of the NPs) and sorted for Lin^-^, Sca1^+^, cKit^+^ cells (Supplementary Fig. 8) for analysis of *β-globin* gene editing by deep sequencing. In these cells, we observed 8.81% editing by deep sequencing (Table 2). It is likely that the sustained correction of anemia and persistence of gene editing into the adulthood of treated mice is explained by the prevalence of a population of fetal HSCs that were successfully edited by the single dose of γtcPNA/DNA loaded NPs.

**Table 2:** Deep sequencing analysis of E18.5 HSCs from the bone marrow of γtcPNA/DNA NP treated fetal mice

Cell type	Amplicons sequenced	Number modified	Frequency (%)
Bone marrow HSCs	4707133	414668	8.81

Deep sequencing analysis of the β-globin locus of genomic DNA from Lin^−^Sca1^+^ cKit^+^ E18.5 fetal bone marrow hematopoietic stem cells following E15.5 injection of γtcPNA/DNA NP. The total number of amplicons sequenced and the number of amplicons with modified sequences are listed

As another method of quantification of editing efficiency, we developed and validated a droplet digital PCR (ddPCR) assay (Supplementary Fig. 9) and used it to quantify the percentage of modified *β-globin* gene alleles in total bone marrow as well as an isolated population of lineage depleted hematopoietic progenitor cells (HPCs) in *Hbb*^*th-4*^*/Hbb*^*+*^ mice 15 weeks after in utero γtcPNA/DNA NP treatment. In the total bone marrow cells, ddPCR analysis confirmed an average of ~6% editing, which is consistent with the deep sequencing data, whereas ~10% gene correction was measured in isolated HPCs (Fig. 4d and Supplementary Fig. 10).

## Discussion

Genetic correction during pregnancy could provide treatment or cure of genetic diseases and allow normal fetal development, a possible advantage compared to treatments given after birth. Monogenic diseases that pose the risk of serious fetal, neonatal, and pediatric morbidity or mortality, such as β-thalassemia, are particularly attractive targets for in utero gene editing [reviewed in^43^]. β-thalassemia and other hemoglobinopathies are relatively common, manifest early in life, and can be cured by low levels of functional protein activity. By delivering gene-editing therapies in utero, it is possible to gain access to dividing stem and progenitor cell populations, which can result in propagation of the corrected gene in all progeny cells.

Here we demonstrate that in utero delivery of PLGA nanoparticles loaded with PNA/DNA is a safe and effective means of achieving clinically relevant frequencies of site-specific, non-enzymatic gene editing in a mammalian fetus that results in sustained postnatal alleviation of disease. A single in utero dose of γPNA/DNA NPs given on E15.5 mediated a level of gene editing sufficient to ameliorate the disease phenotype postnatally. We observed increased hemoglobin concentrations into the wild-type range, improved red cell morphology, reduced reticulocyte counts, and decreased extramedullary hematopoiesis. In addition, we found that in utero gene editing conferred a significant survival advantage on the treated mice compared to untreated controls. These findings suggest that in utero gene editing has the potential to be safe and produce a clinical response substantial enough to reduce β-thalassemia-associated morbidity and mortality.

We examined the biodistribution of nanoparticles after in utero administration and found that the highest accumulation of particles three hours after intravenous administration was within the fetal liver. We also found that intra-amniotic administration results in nanoparticle distribution to the fetal lung and gut at gestational ages later than E15.5. Although not used for gene editing in this study, intra-amniotic delivery of nanoparticles may allow for in utero genetic correction of diseases that affect the lung or gut such as cystic fibrosis. Biodistribution at three hours after administration was selected for this study because, based on in vitro measurements, the nanoparticle formulation used for gene editing releases the majority of its nucleic acid contents within this time frame. The distribution of particles at later time-points merits further study and may reveal that the tissue distribution shifts over time, which could open the possibility of editing other developing tissues of interest, such as the fetal brain, which might require particles with longer release profiles. In this regard, altering the polymer composition, size, or surface modifications could also be investigated as means of changing the distribution or nucleic acid release profile of nanoparticles, as we have shown in other studies^44–47^.

Here we chose to use a γPNA and donor DNA pair previously shown to be effective at achieving gene editing and clinical disease improvement in adult mice with β-thalassemia^6^. We elected to deliver these molecules using PLGA, a biodegradable and biocompatible polymer that is known to be safe in humans. In prior work, clinically relevant levels of gene editing were achieved in adult mice after multiple doses of nanoparticles were administered in combination with stem cell factor (SCF). The use of SCF, the CD117 receptor ligand, was shown to increase the percentage of CD117^+^ hematopoietic progenitor cells in S-phase, elevate DNA repair gene expression, and significantly boost the gene editing frequency^6^. During fetal development, however, it is well known that SCF is highly expressed at sites of hematopoiesis, including the yolk sac, fetal liver, and bone marrow^48–50^. High levels of SCF within the fetal liver may create an environment that is particularly amenable to gene editing using our approach. Of note, an editing frequency of ~4% was achieved in total bone marrow cells and 7% in HSCs in adult mice after four doses of nanoparticles and SCF, compared to ~6% editing in total bone marrow and ~10% in progenitor cells after a single in utero injection containing a fraction of the particles used postnatally [at relative total doses of 185 μg NPs (in utero) versus 8 mg NPs (adult)]. These improvements suggest there may be an advantage to gene editing in the fetus because it is possible to access a rapidly cycling population of HSCs within in the fetal liver.

Unlike other gene editing technologies that rely on activity of exogenous nucleases (CRISPR/Cas, TAL effector nucleases and zinc finger nucleases) that can create extraneous double-stranded breaks, PNA-mediated gene editing makes use of endogenous, high fidelity repair pathways, which reduces the risk of error-prone end-joining causing additional mutations. With continuing concern regarding off-target effects of CRISPR/Cas9^51^ and the finding that Cas9 proteins can illicit an adaptive immune response^52^, the safety profile of PNA/DNA editing may be particularly attractive, as avoiding off-target mutations is of exceptional importance during fetal development.

We found that a single treatment of γPNA/DNA nanoparticles resulted in a gene editing frequency of ~6% in total bone marrow cells (and 8.8% in E18.5 Lin^-^, Sca1^+^, cKit^+^ HSCs and 10% is adult HPCs). The fact that gene editing at these frequencies could yield a discernable phenotypic improvement is consistent with transplantation studies in thalassemic mice and humans, in which low numbers of engrafted donor cells are sufficient to correct anemia^53–55^. This finding is attributed to a positive in vivo selection of genetically corrected erythroblasts^56^. For instance, the average red cell half-life is reduced by 50% in humans with β-thalassemia compared to the red cell half-life in average adults^57,58^. Similarly, others have observed a selective advantage of corrected hematopoietic progenitor cells in patients with severe combined immunodeficiency (SCID)-X1 disease who received viral-mediated gene therapy^59–61^.

The relatively short gestation of mice limited us to delivering just one treatment of nanoparticles. Due to the low toxicity of one dose, we speculate that multiple treatments should be possible in humans or mammals with longer gestational periods, which may result in higher gene editing frequencies. For instance, during human fetal development the liver is the main site of hematopoiesis and HSC cycling from about 6 to 22 weeks of gestation^62^, which could allow for weeks of access to the fetal liver HSCs via umbilical vessel cannulation. Treatment at later time points, when the HSCs primarily reside within the fetal BM, could also be therapeutic since the HSCs are still rapidly cycling [reviewed in^63^]. Additionally, PNA-modifications that enhance target binding such as guanidine-G-clamp PNA monomers^64^, could be used to further improve gene-editing efficiencies.

In utero gene editing may hold great promise for treating or curing numerous inherited human genetic diseases. While the work presented here provides a foundation for a clinically translatable approach for site-specific gene editing in utero mediated by γPNA/DNA nanoparticles, further study is warranted to determine the efficacy and safety of this technique in other disease models, including large animals such as sheep and non-human primates. Success in future investigations could provide a compelling rationale for clinical application.

## Methods

### Experimental design

The main goal of our study was to determine if nanoparticles loaded with PNA and donor DNA could be used alleviate signs of anemia after a single-dose in utero administration in a mouse model of human β-thalassemia. The sample sizes of experiments were selected on the basis of previous experience. Data collection was stopped at a priori defined points for all experiments. For all in utero treatment experiments, animals were randomly assigned to IV, IA, or control treatment groups in a blinded manner. For β-thalassemia mouse experiments, end points were selected based on relevant clinical manifestation of disease in the mouse model. All sample measurements were blinded. For the β-thalassemia survival experiment, age-matched untreated mice were randomly assigned at weaning. The number of replicates for each experiment is stated in each figure legend.

### Oligonucleotides

Mini-PEG γPNA monomers were prepared from Boc-(2-(2-methoxyethoxy)ethyl)-L-serine as a starting material by a series of multistep synthetic procedures including reduction, mitsunobu reaction, nucleobase (A, C, G and T) conjugation and then ester cleavage^31^. At each step, the respective product was purified by column chromatography^30^. PNA oligomers were synthesized on solid support using Boc chemistry^31^. The oligomers were synthesized on MBHA (4-methylbenzhydrylamine) resin according to standard procedures of Boc chemistry. A kaiser test was performed at each step to measure complete coupling and double coupling was performed if it was required. The oligomers were cleaved from the resin using an m-cresol/thioanisole/TFMSA/TFA (1:1:2:6) cocktail, and the resulting mixtures were precipitated with ethyl ether, purified by reversed-phase high-performance liquid chromatography (acetonitrile:water) and characterized with a matrix-assisted laser desorption/ionization time-of-flight mass spectrometer^6^. The sequence of γPNA used in this study is H-KKK-JTTTJTTTJTJT-OOO-TCTCTTTCTTTCAGGGCA-KKK-NH_2_. Underlined bases indicate γPNA residues; K, lysine; J, pseudoisocytosine; O, 8-amino-2,6,10-trioxaoctanoic acid linkers connecting the Hoogsteen and Watson–Crick domains of the tcPNA. The single-stranded donor DNA oligomer was prepared by standard DNA synthesis except for the inclusion of three phosphorothioate internucleoside linkages at each end to protect against nuclease degradation (Midland Certified Reagent Company; Midland, TX). The 60 bp donor DNA matches positions 624–684 in β-globin intron 2, with the correcting IVS2-654 nucleotide underlined: 5′-AAAGAATAACAGTGATAATTTCTGGGTTAAGGCAATAGCAATATCTCTGCATATAAATAT-3′.

### PLGA nanoparticle synthesis and characterization

PLGA (50:50 ester-terminated, 0.55–0.75 g dl^−1^, LACTEL absorbable polymers; Birmingham, AL) NPs containing C6 (Sigma; St Louis, MO) or DiD (Thermo Scientific; Rockford, IL) were synthesized using a single-emulsion solvent evaporation technique^17^. C6 or DiD was added to the polymer solution at a 0.2% wt:wt dye:polymer ratio. PNA/DNA and blank PLGA NPs were synthesized using a double-emulsion solvent evaporation technique modified to encapsulate PNA and DNA oligomers^6,65^. PNAs and donor DNAs were dissolved in 60.8 μl DNAse-free water. All nanoparticle batches had 2 nmole mg^−1^ of γPNA and 1 nmole mg^−1^ of donor DNA. The encapsulant was added dropwise to a polymer solution containing 80 mg 50:50 ester-terminated PLGA dissolved in dichloromethane (800 μl), then ultrasonicated (3 × 10 s) to formulate the first emulsion. To form the second emulsion, the first emulsion was added slowly dropwise to 1.6 ml of 5% aqueous polyvinyl alcohol and then ultrasonicated (3 × 10 s). This mixture was finally poured into 20 ml of 0.3% aqueous polyvinyl alcohol and stirred for 3 h at room temperature. Nanoparticles were then thoroughly washed with 20 ml water (3×) and further collected each time by centrifugation (25,644 × *g* for 10 min at 4 °C). Nanoparticles were resuspended in water, frozen at −80 °C, and then lyophilized. Nanoparticles were stored at −20 °C after lyophilization^6^. Blank NPs were loaded with 1 × phosphate-buffered saline and formulated using the double emulsion method described above. Scanning electron microscopy (SEM) was performed using an XL-30 scanning electron microscope (FEI; Hillsboro, Oregon) as previously described^17^. Dynamic light scattering (DLS) was performed to measure the NPs size (hydrodynamic diameter) and surface charge (zeta potential) using a Malvern Nano-ZS (Malvern Instruments, UK). Nucleic acid release was analyzed by incubating particles (2 mg) in 600 μl 1× phosphate-buffered saline in a 37 °C shaker, spinning down and removing supernatant. The nucleic acid content of the supernatant was measured as the absorbance at 260 nm at the indicated time points.

### Mouse models and genotyping

All animal use was in accordance with the guidelines of the Animal Care and Use Committee (IACUC) of Yale University and conformed to the recommendations in the Guide for the Care and Use of Laboratory Animals (Institute of Laboratory Animal Resources, National Research Council, National Academy of Sciences, 1996). Animal protocols were approved by the IACUC of Yale University. C57BL/6 mice were obtained from Charles River Laboratories (Wilmington, MA). The IVS2-654 β-thalassemia mice were obtained from Ryszard Kole, University of North Carolina (Chapel Hill, NC)^38^. Litters from this mouse model were genotyped prior to weaning. Genomic DNA (gDNA) was isolated from tail clippings using the Wizard SV DNA Purification System (Promega; Madison, WI). Genotyping PCR was performed to detect the presence of the human β-globin gene, indicating the mouse has a *Hbb*^*th-4*^*/Hbb*^*+*^ genotype, using a species independent forward primer (complementary to both mouse and human β-globin sequences) and two species dependent reverse primers. Genotyping primers are as follows: forward—5′-CCCTGGGCAGGTTGGTATC-3′; human reverse—5′-AACGATCCTGAGACTTCCACA-3′; and mouse reverse 5′–AGCAGAGGCAGAGGATAGGTC–3′. PCR was performed using high fidelity Platinum TAQ polymerase (Invitrogen; Carlsbad, CA); reaction conditions are as follows: 5.0 μl 10 × HiFi buffer, 3.0 μl MgCl_2_, 1.0 μl dNTPs, 2.0 μl 10 μM forward primer, 1.0 μl 10 μM human reverse primer, 1.0 μl 10 μM mouse reverse primer, 0.8 μl HiFi Taq, 90–400 ng gDNA and remaining volume to 50 μl with dH_2_O. Thermocycler conditions were as follows: 94 °C 2 min, [94 °C 30 s, 55 °C 45 s, 68 °C 1 min] × 35 cycles, 68 °C 1 min, hold at 4 °C. PCR products were run on a 2% agarose gel. The amplicon derived from the mouse reverse primer is 196 bp and the amplicon from the human reverse primer is 508 bp. The presence of bands at both 196 and 508 bp indicates the *Hbb*^*th-4*^*/Hbb*^*+*^ genotype. A single band at 196 bp indicates the *Hbb*^*+*^*/Hbb*^*+*^ genotype. Only heterozygous mice were included in this study.

### In utero NP delivery and imaging

Time dated pregnant mice (8–12 weeks old) between 15–18 days post conception were anesthetized with inhaled isoflurane (3% vol/vol for induction, 2% vol/vol for maintenance). The gravid uterus was exposed through a midline laparotomy incision. For the biodistribution studies, lyophilized fluorescent nanoparticles were resuspended by vortex and water bath sonication in 1× dPBS to a concentration of 9 mg ml^−1^. Intravascular injections were performed at E15.5 and E16.5. A volume of 15 μl of 9 mg ml^−1^ NP suspension was drawn up into a glass micropipette (tip diameter ~60 μm) and injected intravascularly via vitelline vein of each fetus using a pneumatic microinjector (Narishige; Japan). Intra-amniotic injections were performed at E15.5, E16.5, E17.5 and E18.5. A volume of 20 μl of 9 mg ml^−1^ NP suspension was injected directly into the amniotic cavity of each fetus. As a positive control to detect NPs in maternal circulation, 100 μl of 9 mg ml^−1^ C6 NPs were injected intravenously into a mouse pregnant with fetuses at E15.5. Pregnant mice were killed 3 h post DiD PLGA NP injection and fluorescence and x-ray imaging was performed on a Carestream In-Vivo MS FX PRO (Bruker; Billerica, MA). Pregnant mice were also killed 3 h post C6 PLGA NP injection. Fetuses were delivered via cesarean section and washed in PBS. Ex vivo fetal fluorescence stereomicrope imaging was performed on a Leica M80 stereomicroscope (Wetzlar, Germany). Fetuses and maternal organs were fixed overnight in 4% paraformaldehyde (Electron Microscopy Sciences; Hartfield, PA) at 4 °C. The tissues were next dehydrated in 20% sucrose and embedded in Optimal Cutting Temperature (OCT) Compound (Torrance, CA). Frozen 15 μm-thick fetal and maternal liver sections were mounted on glass slides and stained with Hoescht dye. Confocal imaging of the frozen sections was performed on a Zeiss Axio Observer Z1 microscope (Oberkochen, Germany).

For the safety studies, the fetuses of time dated pregnant C57BL/6 females (8–10 weeks old) were injected intravenously at E15.5 as described above with 15 μl of 9 mg ml^−1^ PNA/DNA NPs. Intra-amniotic injections were performed at E16.5; 20 μl of 9 mg ml^−1^ blank NPs or PNA/DNA NPs were injected into the amniotic cavity. Untreated pregnant mice were anesthetized and the gravid uterus was exposed as described above. The fetuses were counted, the uterus was returned to the abdomen, and the midline incision was closed. The number of untreated, intravenously and intra-amniotically treated pups surviving was counted at the time of weaning, 21 days. The weight of injected and untreated control pups was measured for a period of 10 months.

The fetuses of time dated pregnant *Hbb*^*th-4*^*/Hbb*^*+*^ mice (mated with *Hbb*^*th-4*^*/Hbb*^*+*^ males) were injected intravenously as described above with 15 μl of either 9 mg ml^−1^ or 12 mg ml^−1^ PNA/DNA NPs, correlating to doses of 300 mg kg^−1^ or 400 mg kg^−1^, respectively.

### Cytokine array analysis

The fetuses of time dated pregnant C57BL/6 females (8–10 weeks old) were injected intravenously at E15.5 as described above with 15 μl of blank NPs (9 mg ml^−1^), PNA/DNA NPs (9 mg ml^−1^), or 1× dPBS. After 48 h, fetal plasma samples were collected. Plasma samples from fetuses receiving PBS, blank NPs, or PNA/DNA NPs and untreated fetuses were submitted to the CytoPlex Core Facility at Yale University. The facility performed luminex based cytokine detection and quantification using the Bio-Plex Pro Mouse Cytokine 23-Plex assay available from Bio-Rad (Hercules, CA).

### Peripheral blood analysis

Mice were anesthetized using open-drop 30% w/v isoflurane in propylene glycol. A volume of 50–100 μl of blood was collected retro-orbitally using heparinized micro-hematocrit capillary tubes (Fisher Scientific; Pittsburgh, PA) and evacuated into heparinized coated tubes containing 5 μl 0.5 M EDTA acid. Complete blood counts were performed using a Hemavet 950FS (Drew Scientific; Oxford, CT) according to the manufacturer’s protocol. A volume of 1–3 μl of fresh blood was smeared onto glass slides and stained with Wright–Giemsa stain (Sigma-Aldrich; St. Louis, MO) for 20 s. Slides were washed in 1× dPBS for 10 min and air-dried. An additional 10 μl of blood was incubated with 3 μl new methlylene blue reticulocyte stain (Sigma-Aldrich; St. Louis, MO) for 10 min after which blood smears were prepared. A cover slip was mounted on air-dried smears with Cytoseal 60 (Thermo Scientific; Rockford, IL). All blood smears were imaged on an Olympus FSX100 microscope. Two individuals independently counted the number of reticulocytes present in 500 cells. The relative reticulocyte count was calculated as the number reticulocytes in 1000 RBCs divided by ten.

### Histology

Spleen images were taken and weights were recorded for *Hbb*^*th-4*^*/Hbb*^*+*^ mice 15–30 weeks post PNA/DNA NP delivery, age-matched untreated *Hbb*^*th-4*^*/Hbb*^*+*^ mice, and wild-type mice. The collected spleens were fixed in 10% neutral buffered formalin and processed by Yale Pathology Tissue Services for H&E staining as well as E-cadherin, CD44, CD71 and CD61 immunohistochemistry. All antibodies were used at a dilution of 1:1000. Spleen sections were imaged on an Olympus FSX100 microscope.

### Fetal bone marrow collection and cell sorting

The bone marrow of untreated and PNA/DNA NP treated *Hbb*^*th-4*^*/Hbb*^*+*^ mice were collected 3 days post NP delivery on E18.5 as previously reported^36^. Freshly isolated fetal bone marrow cells were suspended in ice-cold DMEM + (Dulbecco’s modified Eagle’s medium, 10 mM HEPES, 2% fetal bovine serum [FBS]) at 10^7^ cells ml^−1^ and stained for 15 min on ice with anti-cKit-PE (eBioscience, Cat #12-1171-82), anti-Sca1-(PerCP)-Cy5.5 (eBioscience, Cat **#**45-5981-82), and FITC-conjugated lineage marker antibodies [CD4 (eBioscience, Cat **#**11-0041-82), CD8 (eBioscience, Cat #11-0081-82), Ter119 (eBioscience, Cat #11-5921-82), Gr-1 (eBioscience, Cat #11-5931-82), and CD45 (eBioscience, Cat #11-0452-82)] (Thermo Scientific; Rockford, IL). All antibodies were used at a dilution of 1:100. Samples were then washed with 10 × volume of HBSS + (Hank’s balanced salt solution, 10 mM HEPES, 2% FBS) and centrifuged at 0.4 × *g* for 8 min at 4 °C. Cell pellets were resuspended in DMEM + and samples were immediately sorted by flow cytometry (BD FACSAria).

### Deep sequencing analysis

Genomic DNA (gDNA) from the bone marrow of adult PNA/DNA NP treated *Hbb*^*th-4*^*/Hbb*^*+*^ mice and age-matched untreated *Hbb*^*th-4*^*/Hbb*^*+*^ mice was collected using the Wizard SV DNA Purification System (Promega, Madison, WI) according to manufacturer’s instructions. gDNA was collected from sorted fetal bone marrow cells using a phenol-chloroform extraction method. Cells were digested overnight in 10 mM Tris-HCl (pH 8), 150 mM NaCl, 20 mM ethylenediamine tetracetic acid and 1% sodium dodecyl sulfate, with proteinase K. Digests were subjected to extraction with phenol/chloroform/isoamyl alcohol followed by re-extraction with choloroform, precipitated with KOAc in EtOH, spun down and dried at room temperature and resuspended in dH_2_O. PCR reactions were performed with high fidelity TAQ polymerase (Invitrogen; Carlsbad, CA). Each PCR tube consisted of 28.2 µL dH_2_O, 5 µL 10 × HiFi Buffer, 3 µL 50 mM MgCl_2_, 1 µL DNTP, 1 µL each of forward and reverse primer, 0.8 µL High Fidelity Platinum Taq Polymerase and 10 µL 40 ng/ml gDNA. Thermocycler conditions were as follows: 94 °C 2 min (94 °C 30 s, 55 °C 45 s, 68 °C 1 min) x35 cycles, 68 °C 1 min, hold at 4 °C. PCR products were purified using the QIAquick PCR Purification Kit (Qiagen; Hilden, Germany). PCR products were prepared by end-repair and adapter ligation according to Illumina protocols (San Diego, CA), and samples were sequenced by the Illumina HiSeq 2500 with 75 paired-end reads at the Yale Center for Genome Analysis. Samples were analyzed as previously described^5^. Briefly, paired-end reads were merged using PEAR (v. 0.9.6)^66^. The merged reads were mapped to the loci of interest using BWA-MEM aligner (v. 0.7.12)^67^. The nucleotide composition for each position in the alignment was obtained with in-house python scripts. To study off-target effects, we looked for the presence of 16 bp k-mers of the donor sequence in the off-target sequencing libraries allowing for one mismatch using BBTools. The primers used for β−globin intron 2 were as follows:^6^ forward primer: 5′-TATCATGCCTCTTTGCACCA-3′; reverse primer: 5′-AGCAATATGAAACCTCTTACATCA-3′. Primers for off-target sites of partial homology were as follows;^6^ forward primer is listed first: Vascular cell adhesion protein precursor 1 (5′-AGATAATTATTGCCTCCCACTGC-3′ and 5′-AATGGAAGGGCATGCAGTCA-3′); Polypyrimidine tract binding protein (5′-CCCAATCCTGAATCCTGGCT-3′ and 5′-CATACTGATGTCTGTGGCTTGA-3′); Protocadherin fat 4 precursor (5′-AAGCTCAAACCTACCAGACCA-3′ and 5′-AGCTGGAAGCTTCTTCAGTCA-3′); Olfactory receptor 266 (5′-CCCTCTGTGGACTGAGGAAG-3′ and 5′-TGATGAGCTACGGGTATGTGA-3′); Syntaxin-binding protein (5′-CAAAAAGCCTTAAGCAAACACTC-3′ and 5′-TCTCTCCCTCAGCATCTATTCC-3′); Muscleblind-like protein (5′-TGTGTTTGTTTATGGATACTTGAGC-3′ and 5′-GCATGCACAATAAAGGCACT-3′); Ceruloplasmin isoform (5′-CATGGGAAACAGTCAAAAGAAA-3′ and 5′-GTAGGTTTCCCCACAGCTT-3′).

### Droplet digital PCR quantification of editing in bone marrow cells and HPCs

Bone marrow was collected from the femurs of *Hbb*^*th-4*^*/Hbb*^*+*^ mice 15 weeks after NP treatment and age-matched untreated *Hbb*^*th-4*^*/Hbb*^*+*^ mice. Hematopoietic progenitor cells (CD3e^-^, CD11b^−^, CD19^−^, CD45R^−^, Gr-1^-^, Ter119^−^) were isolated by magnetic separation using an EasySep^TM^ Mouse Hematopoietic Progenitor Cell Isolation Kit (STEMCELL^TM^ Technologies, Vancouver, CA) according to manufacturer’s instructions. gDNA was extracted from total bone marrow cells or isolated progenitor populations using the Wizard SV DNA Purification System (Promega, Madison, WI) according to manufacturer’s instructions. The concentration of extracted gDNA samples was measured using a QuBit® dsDNA BR assay kit (Invitrogen, Carlsbad, CA) according to manufacturer’s instructions. Up to 80 ng of gDNA was used for each sample per reaction. PCR reactions were set up as followed: 11 μl 2 × ddPCR™ supermix for probes (no dUTP) (Bio-Rad, Hercules, CA), 0.2 μl forward primer (100 μM), 0.2 μl reverse primer (100 μM), 0.053 μl β-thal probe (100 μM), 0.053 μl wild-type probe (100 μM) (Integrated DNA Technologies, Coralville, IA), 0.5 μl EcoR1, 10 μl gDNA and dH_2_O. Droplets were generated using the Automated Droplet Generator (AutoDG™) (Bio-Rad). Thermocycling conditions were as follows: 95 °C 10 min, (94 °C 30 s, 55.3 °C 1 min – ramp 2 °C/s) x 40 cycles, 98 °C 10 min, hold at 4 °C. Droplets were allowed to rest at 4 °C for at least 30 min after cycling and were then read using the QX200™ Droplet Reader (Bio-Rad). Data were analyzed using QuantaSoft™ software. Data are represented as the fractional abundance of the wild-type allele. The primers used for ddPCR were as follows: forward: 5′-ACCATTCTAAAGAATAACAGTGA-3′, reverse: 5′-CCTCTTACATCAGTTACAATTT-3′. The probes used for ddPCR were as follows: wild-type (FAM): 5′-TGGGTTAAGG**C**AATAGCAA-3′, β-thal (HEX): 5′-TCTGGGTTAAGG**T**AATAGCAAT-3′.

### Statistical analysis

The data are means ± s.e.m. unless otherwise noted and compared using one-way or two-way ANOVA with repeated measures when appropriate. Bonferroni correction was used to correct for multiple comparisons. Survival data were analyzed using a Log-rank test. Statistical analyses were carried out using GraphPad Prism. A *P* value of less than 0.05 was considered statistically significant.

### Data availability

Additional data and movies are available in the supplementary material. Deep sequencing data has been deposited in the NCBI Sequence Read Archive under accession number SRP142526.

## Electronic supplementary material

Supplementary Information

Description of Additional Supplementary Files

Supplementary Movie 1

Supplementary Movie 2

Supplementary Movie 3
