# Supplementary material for: In utero nanoparticle delivery for site-specific genome editing
Source: Nat Commun. 2018 Jun 26;9:2481. doi: 10.1038/s41467-018-04894-2 (PMC6018676; doi:10.1038/s41467-018-04894-2)
Supplement: Supplementary file 1 — Supplementary Information [file 41467_2018_4894_MOESM1_ESM.pdf]

Supplementary Information for

In utero nanoparticle delivery for site-specific genome editing

Ricciardi et al.

This file includes:

Supplementary Figures 1-10

Other Supplementary Materials for this manuscript includes the following:

Supplementary Movies 1-3

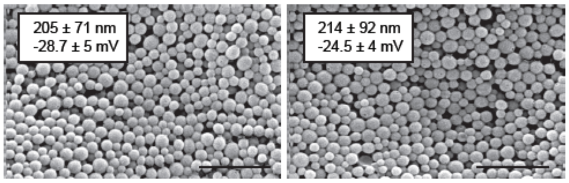

Supplementary Figure 1. Scanning electron microscope (SEM) images of PLGA NPs encapsulating either C6 (left) or DiD (right). Size and zeta potential were measured by dynamic light scattering (DLS), data are mean ± s.d. Scale bar = 1 μm.

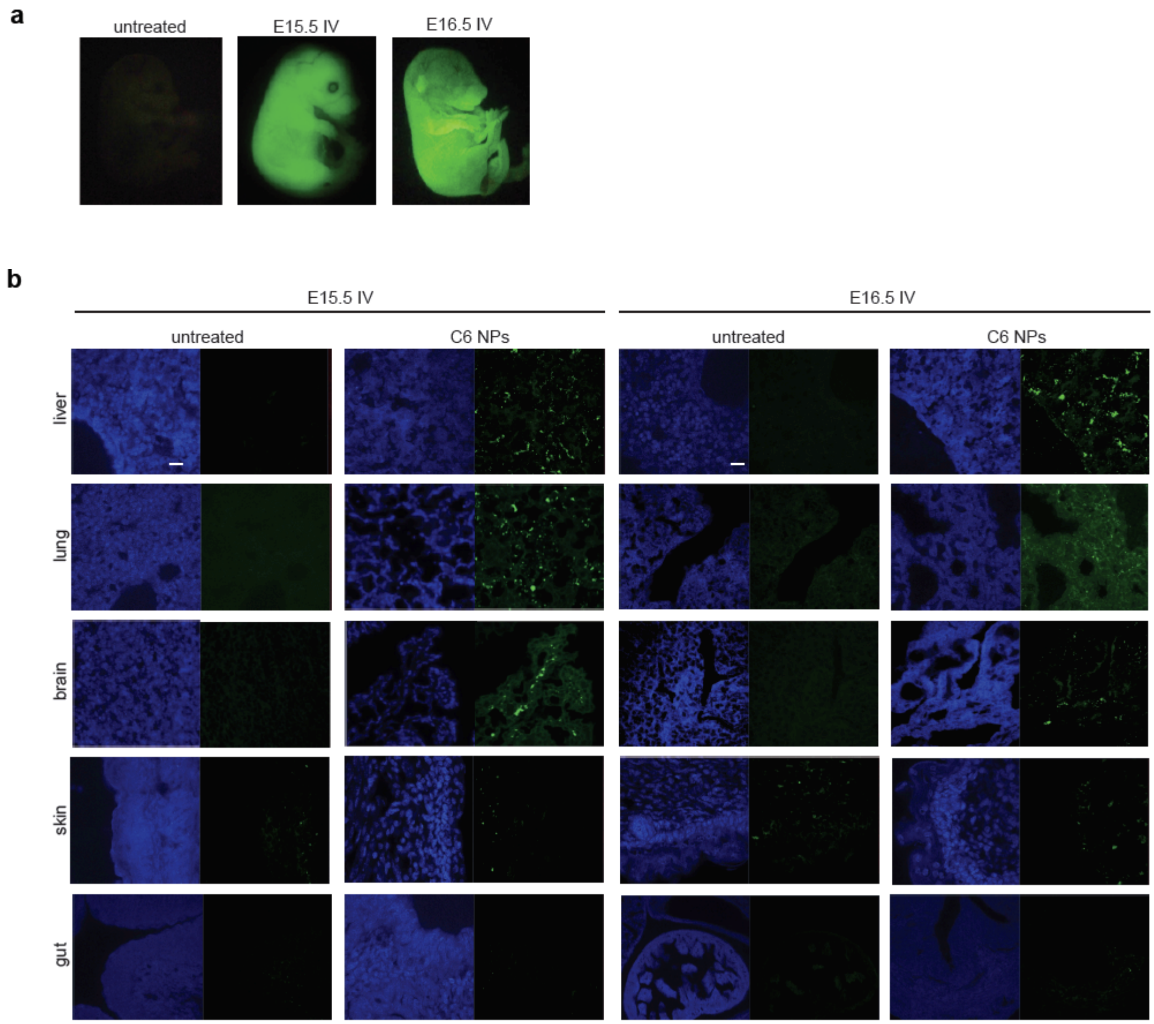

Supplementary Figure 2. **a,** Stereomicroscope and **b,** confocal images of the distribution of C6 PLGA nanoparticles (NPs, green) 3h after intravenous (IV) injection of NPs to fetuses at E15.5 (left) and E16.5 (right) with images of uninjected age matched controls to the left (n=69 fetuses IV), nuclei within the tissues were stained with Hoescht (blue), scale bar = 10 µm.

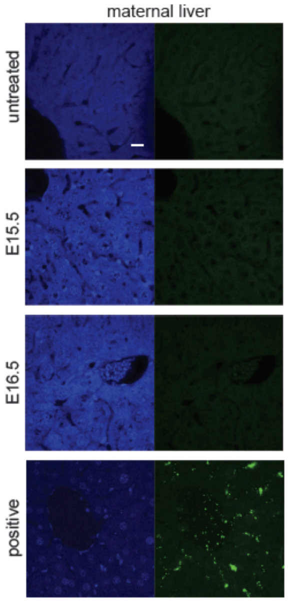

Supplementary Figure 3. Confocal images of maternal livers 3h post intravenous (IV) fetal coumarin 6 (C6) PLGA nanoparticle (NP) delivery; as a positive control, C6 PLGA NPs (green) were directly administered to the maternal circulation of a mouse pregnant with fetuses at E15.5, nuclei within the tissues were stained with Hoescht (blue), scale bar = 10 µm.

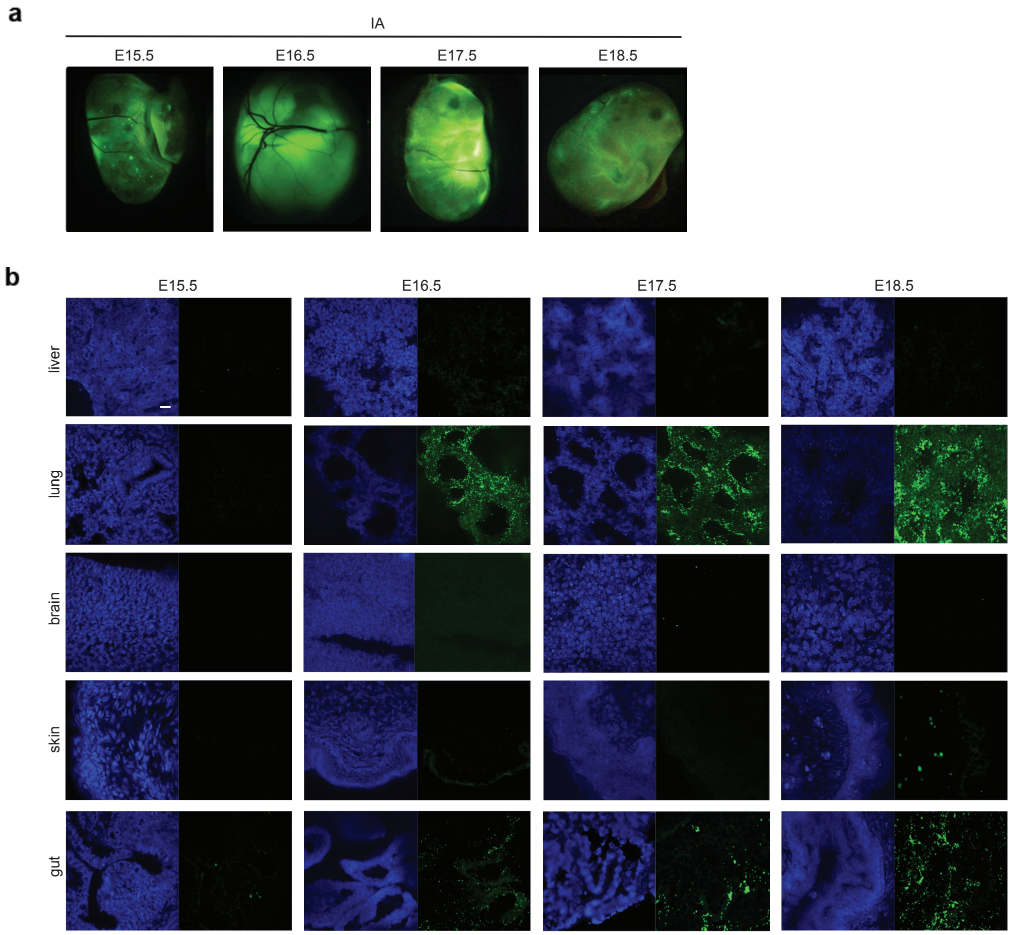

Supplementary Figure 4. a, Stereomicroscope and **b,** confocal images of fetuses 3h after intra-amniotic (IA) coumarin 6 (C6) PLGA nanoparticle (NP, green) delivery from E15.5 to 18.5 (n=140 fetuses IA), nuclei within the tissues were stained with Hoescht (blue), scale bar = 10 μm.

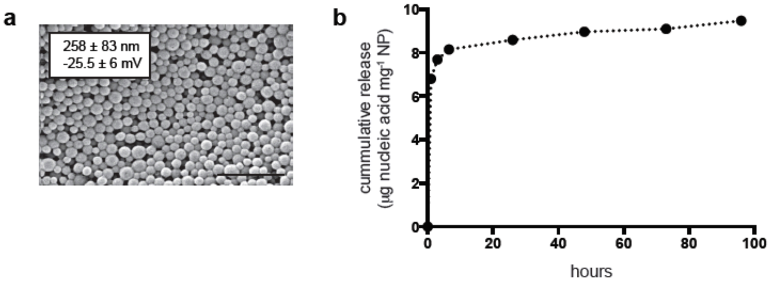

Supplementary Figure 5. a, Scanning electron microscope (SEM) image of γPNA/DNA nanoparticles (NPs). Size and zeta potential were measured by dynamic light scattering (DLS), data are mean ± s.d. Scale bar = 1 µm. **b,** Nucleic acid release profile of γPNA/DNA NPs.

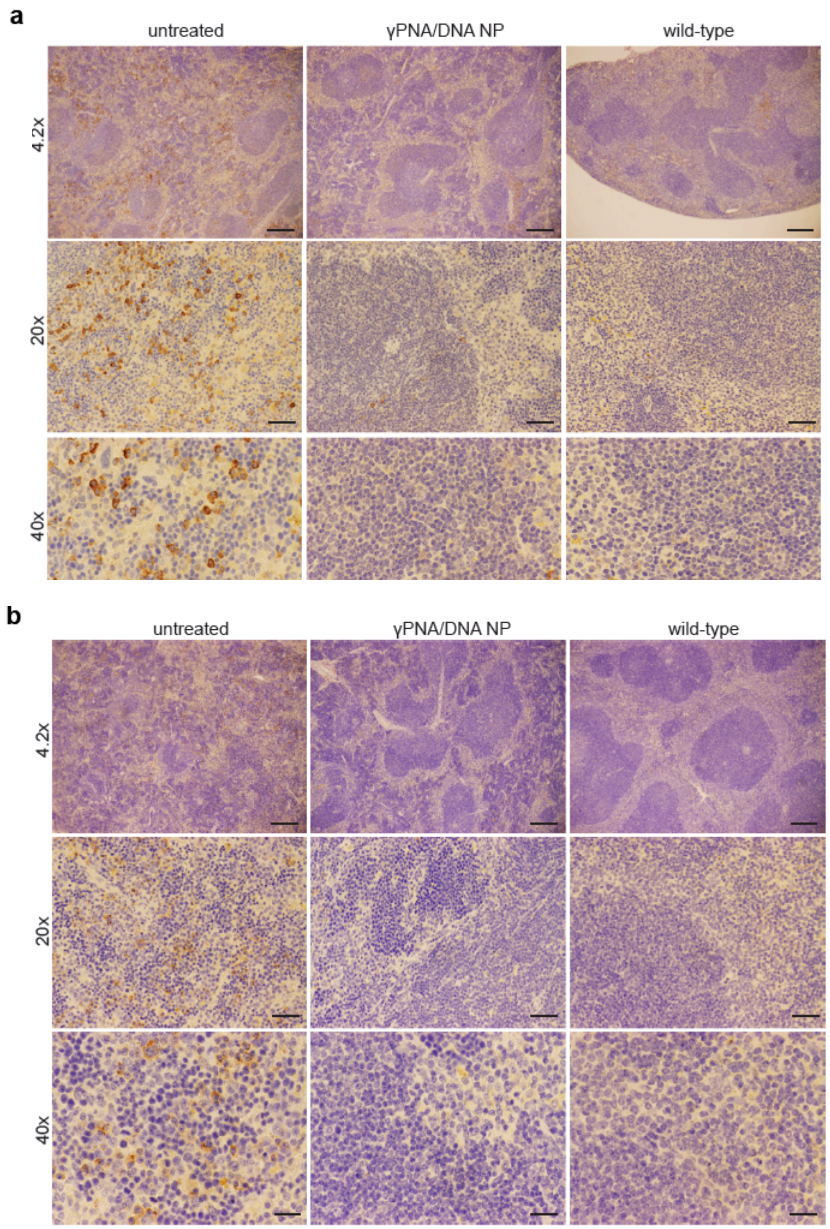

Supplementary Figure 6. Spleen immunohistochemistry stains for **(a)** CD71 and **(b)** CD44. 4.2x scale bars = 150 µm, 20x scale bars = 30 µm, 40x scale bars = 15 µm.

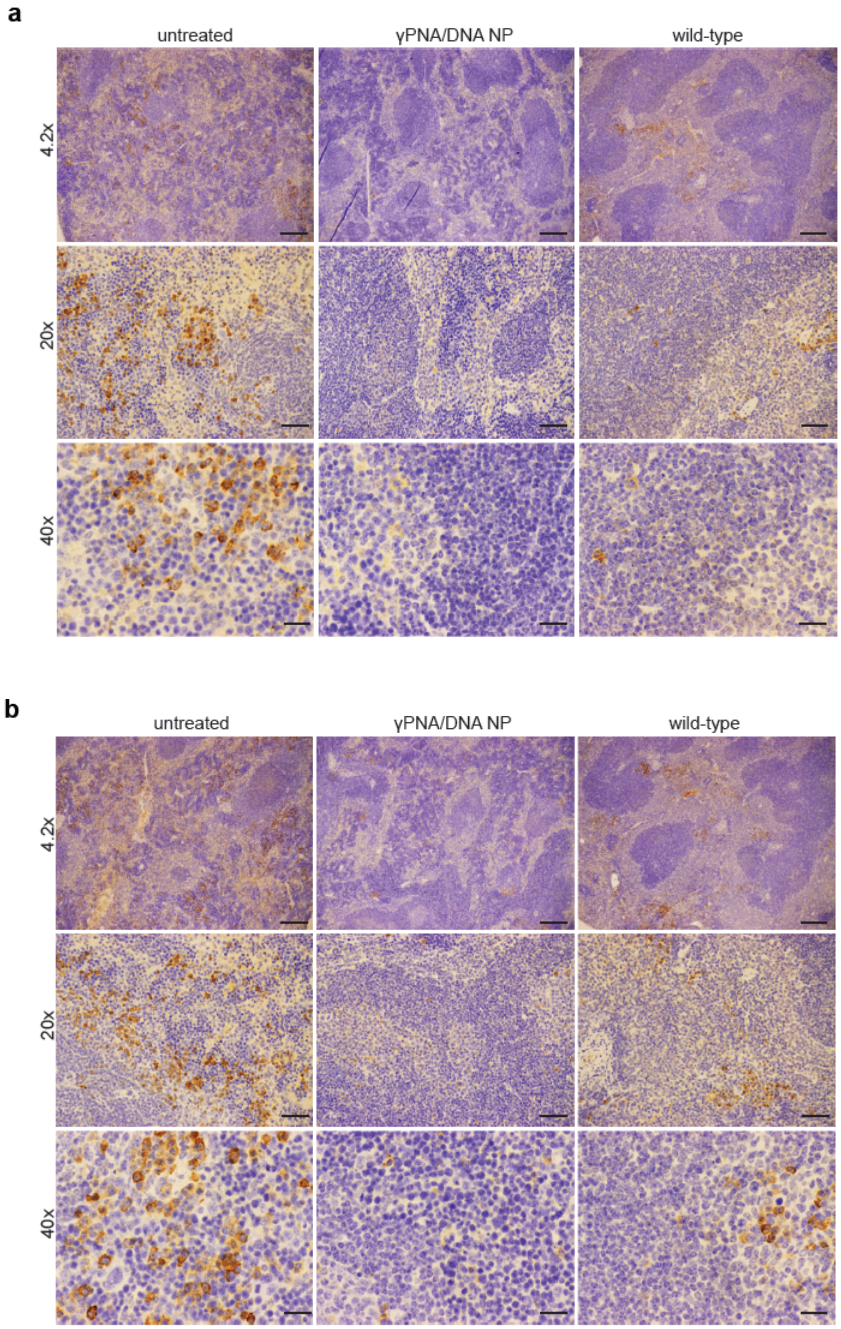

Supplementary Figure 7. Spleen immunohistochemistry stains for **(a)** E-cadherin and **(b)** CD61. 4.2x scale bars = 150 μm, 20x scale bars = 30 μm, 40x scale bars = 15 μm.

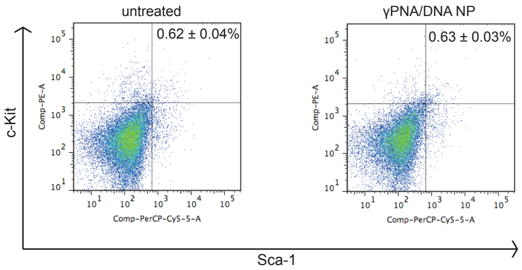

Supplementary Figure 8. Fluorescence-activated cell sorting (FACS) of E18.5 hematopoietic bone marrow stem cells were characterized by expression of c-Kit and Sca-1 proteins and lack of blood lineage protein expression (CD4, CD8, CD45, Ter119, and Gr-1). Data represent the mean percentage of Lin^-, $c\text{-}Kit^+$, $Sca\text{-}1^+$ cells in the total fetal bone marrow cell population at E18.5 (n=7 for each group).

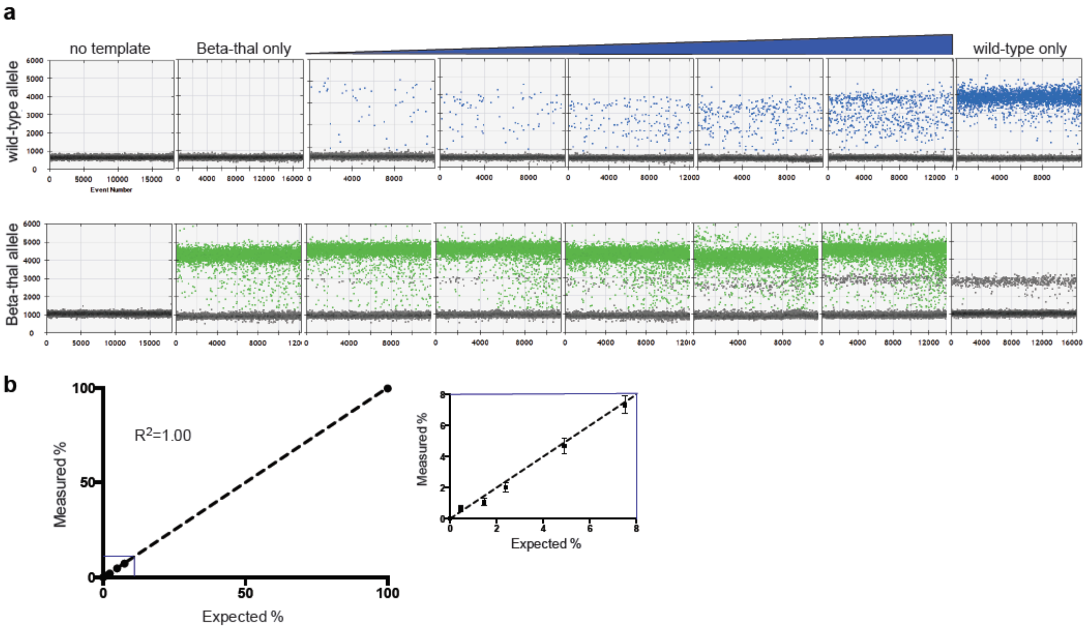

Supplementary Figure 9. Droplet digital PCR (ddPCR) assay validation

a, One dimensional-amplitude plots of beta-thal/wild-type ddPCR assay. A ddPCR assay was designed in which the probes differentiating the two alleles are specific for the gDNA template present in the reaction (beta-thal only and wild-type only controls). Each sample has two plots, one representing the FAM or wild-type allele (blue) and one representing the HEX or beta-thal allele (green). Dots represent individual droplets containing the indicated alleles (droplets containing no gDNA template are gray). The 1D-amplitude plots underneath the blue triangle represent samples in which increasing masses of wild-type gDNA were spiked into samples of beta-thal gDNA.

b, The expected fractional abundance of the wild-type allele (after QuantaSoft™ Software fit the fluorescence data after amplification to a Poisson distribution) was calculated using the ddPCR-quantified copies/µl of wild-type and beta-thal alleles in each control sample. The plot compares the ddPCR measured and expected fractional abundance of the wild-type allele in each sample; the observed correlation is linear. The plot on the right is an expanded view of the lower end of the analysis range, indicated by a blue box on the plot on the left. Error bars indicate the 95% confidence interval.

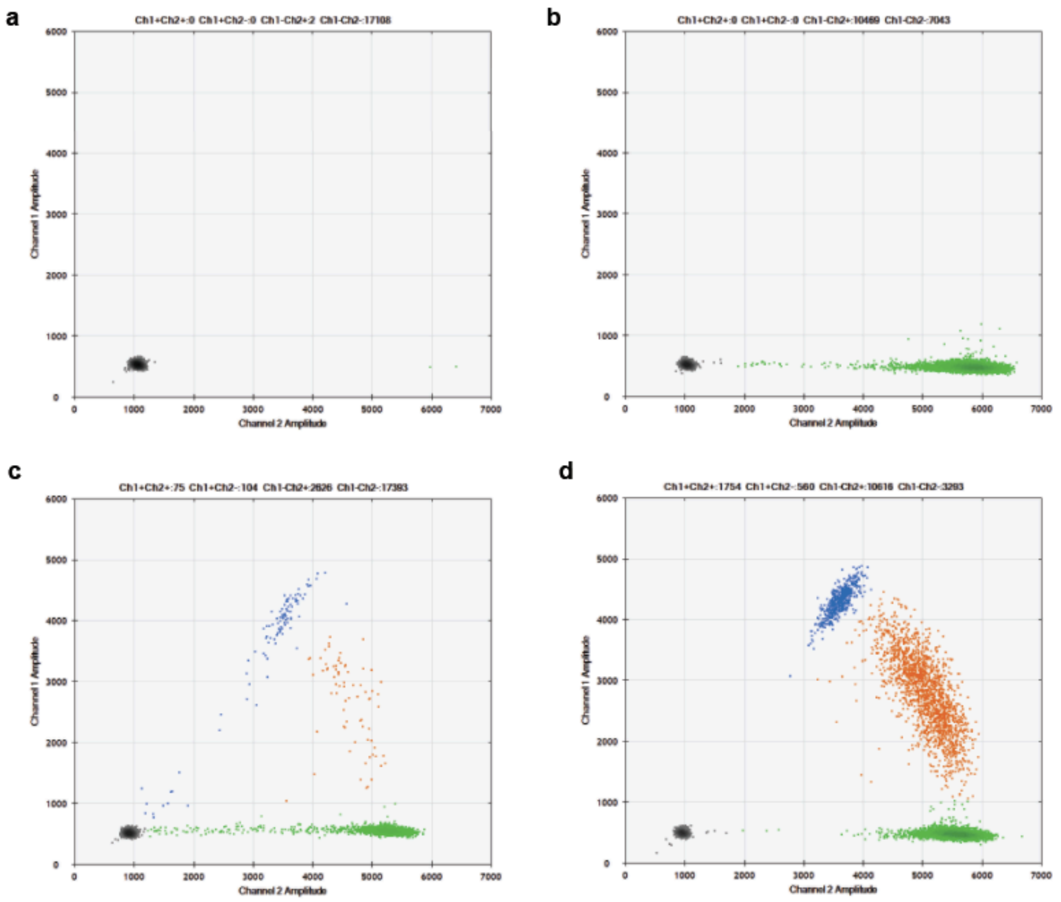

Supplementary Figure 10. Droplet digital PCR (ddPCR) 2D plots of E15.5 intravenous (IV) γtcPNA/DNA nanoparticle (NP) treated bone marrow. Representative 2D ddPCR plots from a no template control **(a)**, genomic DNA (gDNA) from untreated bone marrow genomic **(b)**, and gDNA from total bone marrow **(c)** and an isolated hematopoietic progenitor cell population **(d)** collected from mice 15 weeks post-treatment (E15.5 by IV injection of γtcPNA/DNA NPs). Dots represent individual droplets containing no template (gray), the beta-thal allele (green), the wild-type allele (blue) or both wild-type and beta-thal alleles (orange). The fractional abundance or percent editing of these samples is quantified in Figure 4d.
