# Supplementary material for: In utero nanoparticle delivery for site-specific genome editing
Source: Nat Commun. 2018 Jun 26;9:2481. doi: 10.1038/s41467-018-04894-2 (PMC6018676; doi:10.1038/s41467-018-04894-2)
Supplement: Supplementary file 2 — Description of Additional Supplementary Files [file 41467_2018_4894_MOESM2_ESM.docx]

**Description of Additional Supplementary Files**

File Name: Supplementary Movie 1

Description: Vitelline vein injection of C6 PLGA NPs.

File Name: Supplementary Movie 2

Description: Nanoparticle circulation in an extraembryonic vein (larger vessel) and artery (smaller vessel) 3 hours after vitelline vein injection of C6 PLGA NPs.

File Name: Supplementary Movie 3

Description: Intra-amniotic injection of C6 PLGA NPs.
